# Hepatic barrage to high-flow, intra-hepatic arteroportal fistulas requiring combined interventional approach

**DOI:** 10.1093/bjrcr/uaaf034

**Published:** 2025-07-04

**Authors:** Michele Citone, Antonluca Annese, Giacomo Gabbani, Francesco Pindozzi, Gianmarco Falcone, Emanuele Casamassima, Antonella Santolupo, Silvia Aspite, Lucia Ragozzino, Margherita Falcini, Filippo Biagi, Martina Rosi, Valentina Adotti, Gabriele Dragoni, Davide Roccarina, Tommaso Innocenti, Luca Messerini, Stefano Gitto, Francesco Mondaini, Fabio Marra, Fabrizio Fanelli, Francesco Vizzutti

**Affiliations:** Interventional Radiology Unit, Department of Radiology, Careggi University Hospital, Florence, 50134, Italy; Interventional Radiology Unit, Department of Radiology, Careggi University Hospital, Florence, 50134, Italy; Interventional Radiology Unit, Department of Radiology, Careggi University Hospital, Florence, 50134, Italy; Department of Experimental and Clinical Medicine, University of Florence, Florence, 50134, Italy; Interventional Radiology Unit, Department of Radiology, Careggi University Hospital, Florence, 50134, Italy; Interventional Radiology Unit, Department of Radiology, Careggi University Hospital, Florence, 50134, Italy; Department of Experimental and Clinical Medicine, University of Florence, Florence, 50134, Italy; Department of Experimental and Clinical Medicine, University of Florence, Florence, 50134, Italy; Department of Experimental and Clinical Medicine, University of Florence, Florence, 50134, Italy; Department of Experimental and Clinical Medicine, University of Florence, Florence, 50134, Italy; Department of Experimental and Clinical Medicine, University of Florence, Florence, 50134, Italy; Department of Experimental and Clinical Medicine, University of Florence, Florence, 50134, Italy; Department of Experimental and Clinical Medicine, University of Florence, Florence, 50134, Italy; Gastroenterology Research Unit, Department of Experimental and Clinical Biomedical Sciences, University of Florence, Florence, 50134, Italy; Department of Experimental and Clinical Medicine, University of Florence, Florence, 50134, Italy; Gastroenterology Research Unit, Department of Experimental and Clinical Biomedical Sciences, University of Florence, Florence, 50134, Italy; Pathology Unit, Department of Experimental and Clinical Medicine, University of Florence, Florence, 50134, Italy; Department of Experimental and Clinical Medicine, University of Florence, Florence, 50134, Italy; Interventional Radiology Unit, Department of Radiology, Careggi University Hospital, Florence, 50134, Italy; Department of Experimental and Clinical Medicine, University of Florence, Florence, 50134, Italy; Interventional Radiology Unit, Department of Radiology, Careggi University Hospital, Florence, 50134, Italy; Department of Experimental and Clinical Medicine, University of Florence, Florence, 50134, Italy; Portal Hypertension Departmental Unit, Dipartimento Oncologico e di Chirurgia ad Indirizzo Robotico, Azienda Ospedaliero Universitaria Careggi, Florence, 50134, Italy

**Keywords:** TIPS, IAPFs, portal hypertension, bowel ischaemia, variceal bleeding

## Abstract

Transjugular intra-hepatic porto-systemic shunt (TIPS) is a proven strategy for the management of portal hypertension (PH) complications. Here, we report on a complex case of haemorrhagic shock due to the rupture of gastro-oesophageal varices in the context of PH originally sustained by idiopathic, likely congenital, high-flow intrahepatic arterioportal fistulas (IAPFs) preceded by extensive bowel ischaemia. While the occlusion of the IAPFs potentially controlled the steal of arterial splanchnic blood into the portal circulation, it failed to manage PH related bleeding, necessitating the placement of a salvage TIPS. Porta-caval pressure gradient persisted markedly increased after IAPFs occlusion, indicating an independent intra-hepatic component causing PH. Moreover, hepatic histology demonstrated a pre-sinusoidal/sinusoidal barrage response of the hepatic parenchyma secondary to long-standing IAPFs, causing the onset of an intra-hepatic component of PH. For these reasons, the combined interventional approach led to resolution of the refractory portal hypertensive bleeding, avoiding fatal evolution of diffuse bowel infarction.

## Introduction

In Western countries, non-cirrhotic portal hypertension (PH) accounts for less than 10% of cases of PH.^1^ Intrahepatic arterioportal fistulas (IAPFs) are a rare cause of PH. However, advances in diagnostic techniques have helped to increase their detection. Aetiologies of IAPFs comprise trauma, surgery, trans-hepatic interventions or biopsy, malignancy, ruptured hepatic artery aneurysms, or hereditary haemorrhagic telangiectasia. The clinical manifestations of IAPFs are related to the shunted volume and liver resistance opposed by sinusoids,^2–4^ and patients may be asymptomatic or present with symptoms of PH. Moreover, IAPFs may impair arterial perfusion in the liver and the bowel and the resulting clinical picture, often dominated by PH, may include features of mesenteric steal syndrome.^5^ Importantly, in contrast to systemic arterio-venous shunts, IAPFs rarely have an impact on the cardiac function, because of a damping exerted by the liver interposed in the venous circulation (sinusoidal barrage) determining the pathophysiological consequences of IAPFs on the portal circulation.^6^^,^^7^ Here, we describe a case of life-threatening, PH-related upper digestive haemorrhage and extensive bowel ischaemia due to mesenteric steal, in the context of an atypical, adult-onset idiopathic IAPFs. The patient was successfully managed by sequential endovascular occlusion of the IAPFs and transjugular intra-hepatic porto-systemic shunt (TIPS).

## Case report

**Figure 1.**
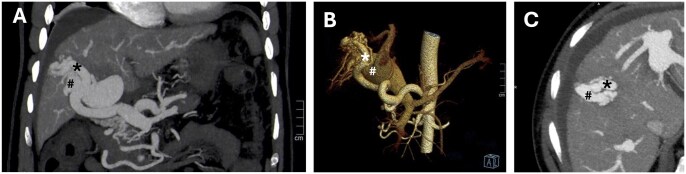
CT scan arterial phase multi-planar reconstruction (A), volume rendering (B) demonstrating intra-hepatic arterio-portal fistulas (hepatic artery branches for liver segment 8 and 5) and severe dilation of the main portal vein. Panel C shows a detailed view of the artery supplying the arteriovenous fistula and its corresponding portal drainage. The feeding artery is marked with an asterisk while the portal venous drainage is indicated by a hashtag.

A 54-year-old male presented to the emergency room with diffuse moderate abdominal pain. His medical history was essentially unremarkable except for long-standing dyspepsia. Blood chemistries were not informative at hospital admission. Physical examination of the abdomen was essentially normal, revealing only a slight increase in entero-colic bloating and a less frequent bowel sounds. There was no evidence of free fluid in the abdomen and no rebound tenderness was appreciated. After an initial spontaneous clinical improvement, the patient experienced worsening abdominal pain, which was associated with leucocytosis (18 × 10^3^/µL), elevated serum lipase (300 U/L), and progressively increasing lactate levels (peak 8 mmol/L). A contrast-enhanced abdominal CT scan revealed the presence of high-flow, type 1 IAPFs^8^ involving segment 8 (Figure 1A–C). Moreover, diffuse splanchnic ischaemia affected the entire digestive tract and the spleen. In the absence of splanchnic venous or arterial thrombosis, or haemodynamically significant arterial stenosis and with stable systemic haemodynamics, ileo-colic ischaemia was attributed to a steal syndrome. This was further aggravated by evidence of reversed blood flow in the portal system including superior mesenteric vein, as demonstrated at ultrasonographic assessment including eco-colour-doppler, consistent with impaired splanchnic venous outflow resulting from the portal hypertensive state and responsible for a concurrent venous passive congestive ischaemia. No findings suggestive of liver cirrhosis were present at imaging, which showed diffuse hypoperfusion of the right hepatic lobe in the arterial phase.

The clinical picture was complicated by the rapid accumulation of moderate-grade ascites. The serum-ascites albumin gradient was greater than 1.1 indicating that the effusion was related to PH. Additionally, there were no signs of spontaneous or secondary bacterial peritonitis.

**Figure 2.**
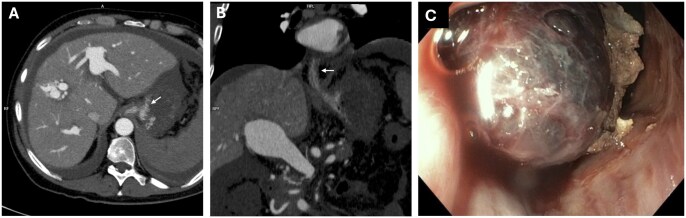
CT scan portal phase showing gastric (A, axial) and oesophageal varices (B, coronal). Upper endoscopy demonstrating a large bleeding gastro-oesophageal varices type 1 treated with cyanoacrylate injection (C).

**Figure 3.**
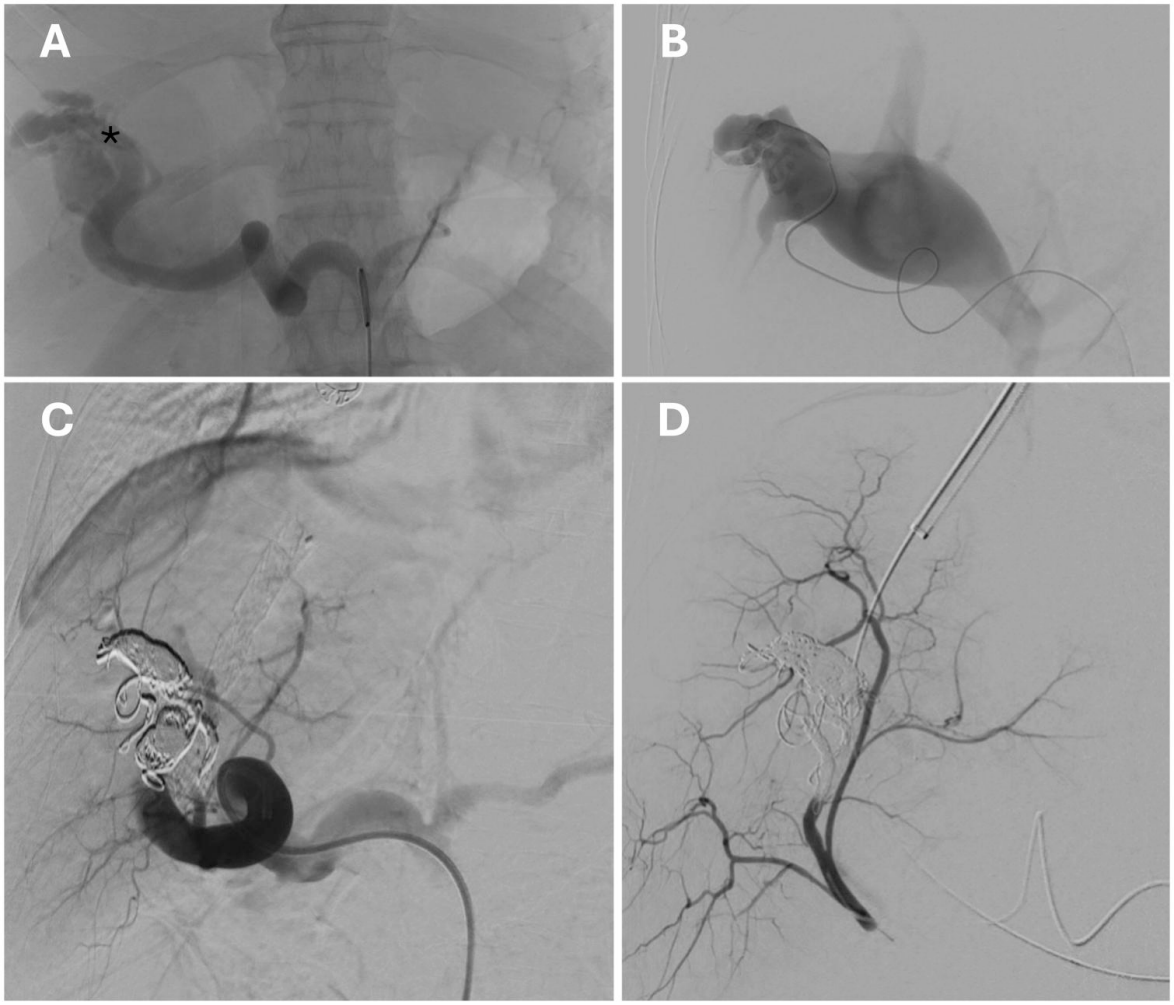
High flow type 2 intra-hepatic arterio-portal fistulas demonstration by selective arteriogram. Enlarged portal vein was immediately opacified with no enhancement of downstream hepatic artery branches (A and B). In panel A, the arterial branch supplying segments V-VIII is marked with an asterisk. Occluded intra-hepatic arterio-portal fistulas with no enhancement of portal vein by super-selectively injecting contrast dye into branches of the hepatic artery (tributary of eighth and fifth liver segment) feeding the IAPFs (C). Following intra-hepatic arterio-portal fistulas occlusion downstream hepatic artery branches for segments 8 and 5 were enhanced.

The patient was therefore scheduled for emergency endovascular occlusion of the IAPFs, but the clinical scenario rapidly deteriorated due to massive haematemesis and enterorrhagia, leading to severe anaemia and hypovolaemic shock. An abdominal CT scan excluded that intestinal ischaemia had evolved into transmural infarction. An upper endoscopy showed clots in the middle and distal portions of the oesophagus and a large clot occupying nearly the entire stomach, from the gastric fundus to the pyloric region. After removing the clots using a loop and a net, and performing extensive washing with saline solution, large oesophageal varices with cherry red spots and an actively bleeding type 1 gastro-oesophageal varice (GOV1) were observed (Figure 2). Bleeding was subjected to emergency treatment with cyanoacrylate injection into the oesophageal side of the GOV1, resulting in the apparent cessation of bleeding. Nonetheless, the gastric cavity was not adequately explored due to the persistence of large clots firmly adhering to the gastric wall. The procedure was therefore halted to allow the patient, mechanically ventilated and under high-dose inotropic vasoactive support, to be transferred to the angiography suite for embolization of the IAPFs. Prior and during the interventional procedures the patient received a total of 6 units of packed red blood cells, intravenous somatostatin at a dose of 500 mcg/h following a 500-mcg bolus, and prophylactic antibiotic therapy with ceftriaxone. Indeed, a further anaemization and fresh blood retrieval from the naso-gastric tube were observed during the interventional procedure. In the angiography suite, a transjugular intrahepatic portal vein catheterization (Rosh Uchida Transjugular liver access set, COOK Medical, Bloomington, IN, USA) was first performed using combined real-time ultrasound and fluoroscopy guided access of the main right portal vein branch, confirming the presence of an elevated portal pressure (PP, 35.5 mm Hg) and porto-caval pressure gradient (PCPG, 32.3 mm Hg). Thereafter, the main hepatic artery was catheterized accessing from the right femoral artery, followed by super-selective catheterization of the branch supplying segment 8 and subsequently segment 5. Angiography confirmed the presence of multiple high-flow IAPFs, indicated by rapid contrast flow into the portal system (Figure 3) and significantly reduced arterial perfusion of the tributary hepatic parenchyma. Embolization of the arterial branches, namely from the 8 and the 5 segment, supplying the fistulous tracts was then performed using controlled-release coils (Penumbra Ruby and Packing coils, Alameda, CA, United States) (Figure 3).

**Figure 4.**
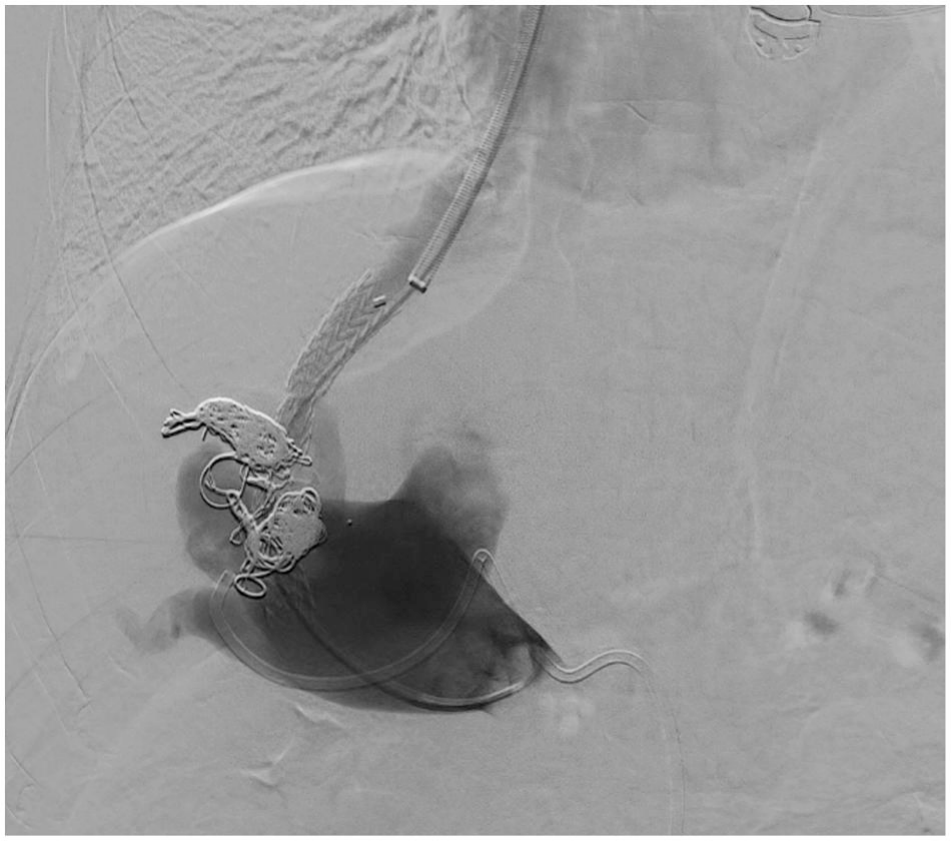
Phlebography of the portal venous system following TIPS positioning and confirming adequate systemic derivation of contrast dye. The intra-parenchymal portion of the endoprosthesis was under-dilated to 6 mm.

After achieving the disconnection of the IAPFs no reduction in the PP and PCPG (37.2 and 34 mm Hg, respectively) was observed. The slightly increased PCPG compared to baseline values, was likely due to the improvement in systemic haemodynamics obtained during the intervention. Thus, exclusion of high-flow IAPFs was not effective in controlling the severe portal hypertensive state. As the persistence of an elevated PCPG supported an intra-hepatic mechanism responsible for PH, we decided to proceed with the creation of a salvage TIPS. The concern for the possible development of a high-output heart failure potentially complicating TIPS was considered unlikely after occlusion of the IAPFs. Therefore, a Viatorr CX 7 + 2 cm endoprosthesis (Gore, Flagstaff, Ariz, United States) was deployed (Figure 4) between the right hepatic vein in the inferior vena cava and the main portal vein right branch. The trans-parenchymal tract was pre-dilated with a non-compliant 6 mm angioplasty balloon catheter (Dorado, BD, San Diego, CA, United States) and the endoprosthesis was post-dilated with the same device along the intra-parenchymal tract, including both the wall of the portal vein and the hepatic vein, thus obtaining an intentional stenosis to reduce the likelihood of developing post-derivative complications. The PCPG fell to 7.9 mm Hg (about 70% lower than the baseline value), indicating an adequate haemodynamic result. Therefore, no further dilation of the endoprosthesis was performed. Importantly, the porto-systemic collaterals supplying GOV identified prior to the opening of the shunt were no longer visible at portal phlebography. Prior to TIPS deployment, a transjugular liver biopsy was performed (2 passages on different liver segments) to define the underlying pathogenic mechanism responsible for the elevated PCPG values.

The patient’s clinical condition subsequently rapidly stabilized, with resolution of clinical signs and symptoms related to PH and intestinal ischaemia. The subsequent follow-up, currently 13 months post-IAPFs occlusion and TIPS, was uneventful, and no complications from the derivative procedure such as congestive heart failure or hepatic encephalopathy were observed. Additionally, no clinical signs compatible with short bowel syndrome or intestinal obstruction due to strictures, as a consequence of intestinal ischaemia, were reported. The hepatocellular function remains excellent.

**Figure 5.**
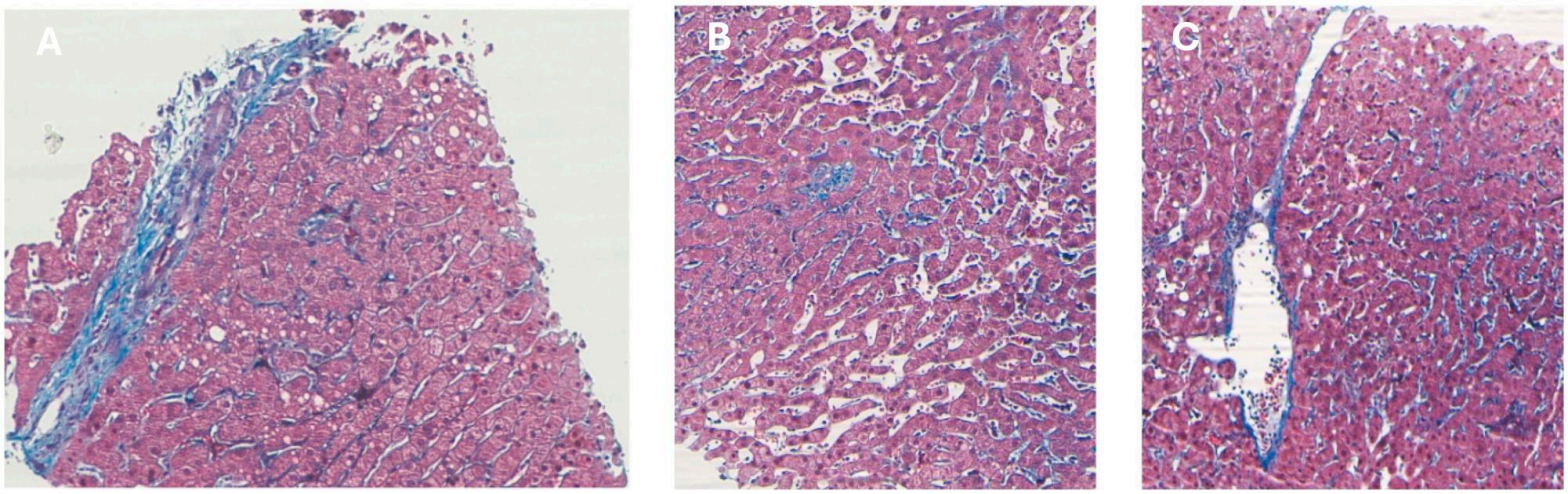
Histological findings: (A) portal vein and portal space fibrosis (Masson trichrome × 25); (B) sinusoidal dilatation and mild sinusoidal fibrosis (Masson trichrome × 10); and (C) mild fibrosis of the central vein (Masson trichrome × 10).

The histopathological examination (overall 32 mm long specimen, 16 complete portal tracts) (Figure 5) demonstrated the presence of periportal vein and pericentral fibrosis, increased number of small portal venous roots, and sinusoidal dilation with mild peri-sinusoidal fibrosis. This picture is suggestive of a progressively developed barrage mechanism resulting from marked portal in-flow secondary to the high-flow IAPFs. Histology confirmed the absence of liver cirrhosis and other conditions possibly responsible for persistence of PH despite IAPFs occlusion.

After TIPS placement, no thrombotic complications occurred, despite the marked reduction of portal blood flow following IAPFs exclusion in the context of pronounced ectasia of the trunk and intra-hepatic branches of the portal vein. As a prophylactic measure, low molecular weight heparin (4000 IU/day) was administered during the first 3 months, then discontinued after confirming the stability of the clinical and biochemical profile and a marked reduction in portal vein diameter (from 41 to 28 mm) was observed.

## Discussion

The portal venous system is peculiarly characterized by high volume, low resistance, and slow flow. PH therefore develops when there is an increase in blood flow, or an increase in resistance opposed to portal blood flow, or when both mechanisms coexist. IAPFs represent a rare cause of PH. Idiopathic IAPFs related to congenital vascular abnormalities are rarely a cause of PH,^9^ and clinical manifestations in adulthood are only occasionally observed. Despite an accurate collection of patient’s clinical history, we were unable to identify potential mechanisms that could have led to decompensation of a condition that is likely to have developed over decades.

The case presented herein was characterized by severe, potentially life-threatening complications of idiopathic IAPFs, and their management may not be straightforward when PH related gastrointestinal bleeding is present.^10^ Indeed, whether to directly treat the IAPFs or to perform a portal systemic shunt depends on the prevalent underling pathogenetic mechanism, that is, increased portal flow versus increased hepatic vascular resistance, respectively. In this case, a treatment modality limited to occluding the IAPFs was ineffective in controlling the bleeding, and on the other hand, the exclusive positioning of TIPS would have been likely inadequate in managing intestinal ischaemia. Additionally, TIPS as a single procedure would have exposed the patient to a considerable risk of high-output cardiac failure. After the exclusion of the IAPFs, the PCPG of this patient remained elevated, because the liver had developed a pre-sinusoidal/sinusoidal barrage, counteracting the high portal vein inflow and limiting the cardiac volume overload, in contrast to what occurs in cases of systemic arterio-venous fistulas. Histopathological examination confirmed that changes secondary to increased portal inflow resulted in development of an intra-hepatic PH, which required the concurrent placement of a TIPS. Importantly, to the best of our knowledge, shunt treatment has only been reported in the management of cases with diffuse arteriovenous fistulae,^11^ without, however, describing the patient’s cardiac outcome.

## Learning points

- This report describes for the first time the successful sequential application of interventional techniques in the treatment of complications of adult-onset idiopathic intrahepatic arterioportal fistulas (IAPFs), guided by portal pressure monitoring.
- Our approach allowed the control of portal hypertension-related bleeding caused by the development of hepatic sinusoidal barrage to IAPFs, and the reversal of extensive intestinal ischaemia.

## References

[1] Berzigotti A , Seijo S, Reverter E, Bosch J. Assessing portal hypertension in liver diseases. Expert Rev Gastroenterol Hepatol. 2013;7:141-155. 23363263 10.1586/egh.12.83

[2] Eastridge BJ , Minei JP. Intrahepatic arterioportal fistula after hepatic gunshot wound: a case report and review of the literature. J Trauma. 1997;43:523-526. 9314320 10.1097/00005373-199709000-00024

[3] Strodel WE , Eckhauser FE, Lemmer JH, Whitehouse WM Jr, Williams DM. Presentation and perioperative management of arterioportal fistulas. Arch Surg. 1987;122:563-571. 3555408 10.1001/archsurg.1987.01400170069010

[4] Vauthey JN , Tomczak RJ, Helmberger T, et al The arterioportal fistula syndrome: clinicopathologic features, diagnosis, and therapy. Gastroenterology. 1997;113:1390-1401. 9322535 10.1053/gast.1997.v113.pm9322535

[5] Capron J-P , Gineston J-L, Remond A, et al Inferior mesenteric arteriovenous fistula associated with portal hypertension and acute ischemic colitis. Successful occlusion by intraarterial embolization with steel coils. Gastroenterology. 1984;86:351-355. 6690362

[6] Graham WP , Eiseman B, Pryor R. Hepatic artery aneurysm with portal vein fistula in a patient with familial hereditary telangiectasia. Ann Surg. 1964;159:362-367. 14129379 PMC1408598

[7] Fulton RL , Wolfel DA. Hepatic artery-portal vein arteriovenous fistula. Arch Surg. 1970;100:307-309. 5308852 10.1001/archsurg.1970.01340210083021

[8] Norton SP , Jacobson K, Moroz SP, et al The congenital intrahepatic arterioportal fistula syndrome: elucidation and proposed classification. J Pediatr Gastroenterol Nutr. 2006;43:248-255. 16877994 10.1097/01.mpg.0000221890.13630.ad

[9] Nookala A , Saberi B, Ter-Oganesyan R, Kanel G, Duong P, Saito T. Isolated arterioportal fistula presenting with variceal hemorrhage. World J Gastroenterol. 2013;19:2714-2717. 23674881 10.3748/wjg.v19.i17.2714 PMC3645392

[10] Kumar A , Ahuja CK, Vyas S, et al Hepatic arteriovenous fistulae: role of interventional radiology. Dig Dis Sci. 2012;57:2703-2712. 22875308 10.1007/s10620-012-2331-0

[11] Aithal GP , Alabdi BJ, Rose JD, James OF, Hudson M. Portal hypertension secondary to arterio-portal fistulae: two unusual cases. Liver. 1999;19:343-347. 10459634 10.1111/j.1478-3231.1999.tb00059.x

